# Discussions of the Kentucky State Dental Association

**Published:** 1860-07

**Authors:** 


### Proceedings of Societies.

### DISCUSSIONS OF THE KENTUCKY STATE DENTAL ASSOCIATION.

THE first topic taken up, was Filling Teeth.

Dr. DRIGGS said that he had some experience in filling teeth with the material known as Osteoplastic; it had not met either his expectations or his desires; this material did not harden with sufficient rapidity; he had found various results from different manipulations; used Pearson's.

Dr. PECKOVER about a year ago got a bottle of Pearson's, but it would not harden except by shaking and drying the superfluous moisture with bibulous paper. He saw some fillings a short time ago, very good; had lately bought some of Roberts' Os-artificial, which does much better; sets quicker and very hard; so rapidly did it harden that it spoiled a burnisher the second day with which he tried to burnish it; there was certainly a great difference between the two materials; one sets in five or ten minutes, the other as quickly as it can be introduced.

Dr. DAWES had a tooth filled with os artificial, the nerve of which was destroyed; for a few days it was very painful; so much so that for two or three days he thought inflammation would ensue; but the pain has entirely subsided since, and it feels very comfortable, and is very hard; it solidified in a few minutes.

Dr. McCLELLAND remarked in reference to Dr. Dawes' case that he would not attribute it to the kind of filling; any other filling would have produced the same result; all dead teeth have some secretions, and will produce the same sensations, even when filled closely with cotton.

Dr. PECKOVER said a gentleman called to have a tooth filled; he had destroyed the nerve and prepared fangs; drilled out fangs, filled with os artificial, expecting to cut it out and fill with gold, and when requested to return and have it removed and gold put in, the patient objected and said the os artificial was so satisfactory that he did not want to change.

Dr. E. J. PECKOVER said he had several fillings in his own family of Pearson's osteoplastic; it did not harden rapidly, but by the use of bibulous paper it got hard in a few days, and the patients were doing well.

Dr. GRANT had used Pearson's osteplastic; had expected much, but was disappointed; it did not get hard as rapidly as he expected; had attempted some difficult filling, but failed to produce satisfactory results.

Dr. FOOTE had some experience; impressions were unfavorable; where the tooth was good, he must object to putting it in, because of the caustic qualities of the material, as it would likely injure the tooth bone; was opposed to the use of caustics; in some cases he observed a yellow tinge around the fillings; attributed this to the caustic effects of the materials; preferred for temporary filling, Hills' stopping or Bevin's filling; for permanent work—gold.

Dr. GRANT has had some experience in the use of chloride of zinc; had been very cautious; used it in adult teeth with great success in allaying pain and without injurious effects; should be used with great caution, otherwise injury will result; lets it remain from five to thirty minutes.

Dr. TALBERT related his experience with chloride of zinc and filled with crystal gold, and found after a year that it was necessary to remove it and replace the filling, the bone having disintegrated; attributed it to the chrystal gold, rather than to zinc; he had found in cavities much sensitiveness and on application of the zinc for a few moments, the sensitiveness subsided, and the tooth was filled with comfort.

Dr. JUSTICE related a similar experience and confirmed Dr. Talbert's conclusion. The objections to using caustics in the teeth of persons of fifteen years of age or under, is that the tooth bone is less dense, and therefore will absorb the caustic more rapidly.

Dr. FOOTE drew the same conclusions from similar reasons, and because the circulation is greater and the recuperative powers better, and nature more rapidly repairs any mischief that may be done.

Dr. ROGERS had not an extensive experience but had watched with much care, all the information that could be gained through the journals, and from experience; used it with much caution, only in superficial cavities, and none with perfect success; he first rubs the tooth with wax; uses crystalized chloride generally but sometimes the deliquesced article; thinks that chloride of zinc in chloroform can be preserved without deliquescing; was very cautious, generally followed with carbonate of soda, and cut with sharp excavators, so as to remove all that was touched; generally prefers to depend upon a sharp instrument, and the firm resolution of the patient.

Dr. STONE thought that in shallow cavities, a small, sharp, keen bur drill, like the French, will remove the sensitiveness, with less pain, and in less time, than with caustics.

Adjourned.

SECOND DAY—MORNING SESSION.

*First Day's Topic continued.*

Dr. FOOTE made a few remarks on the use of the mallet, giving Dr. Atkinson, of Cleveland, credit for originality as well as practical illustrations and teachings, by filling a tooth in the mouth of Dr. Taft, filling fangs as well as tooth; the description being the same as that published in the April number of the *Register.*

Dr. ROGERS: can advance nothing new. Take first molar, decayed on anterior approximal side, complicated with coronal decay, cuts out cavity square into the coronal cavity; cuts gold into strips, and folds in on regular long strips, cuts into proper length, and on a watch maker's broach formed into cylinders; takes one of these cylinders, thrusts it into one side of the bottom of cavity, then one on the opposite side, leaving a space, then another between, projecting beyond the margin of the cavity, bringing up into an angle at the point of reunion of approximate and coronal cavities, then consolidate as a finished filling; then add to that enough to complete the filling, bringing all pressure as nearly in a line with the axis of the tooth as possible; in using cylinders, unless the gold is adhesive, the lamina should stand erect, otherwise after wear, the indentations of instruments would show, by the scaling off of crushed portions; does not anneal cylinders, nor frequently strips, unless some particular object is to be obtained; in very shallow, or superficial cavities does not use cylinders, but where there is any reasonable depth does; has made some good fillings of crystal foil, and some failures; thought it depended upon the character of the operation.

Dr. MCCLELLAND described his method in proxmate cavities in bicuspids; used as traight chisel beveled from one side, can do it with more rapidity and less pain. After removing the edge and giving sufficient room, with file, we smooth the edge, and with a large sharp excavator, removes the principal decay; then well prepare the cavity, near the gum, shaping it as near square as practicable, uses gouges and spoon-shaped excavators, straight or right and left as best suited to reach the point; cuts shoulders or pockets in the side, so as to get a firm hold, using a hatchet, bending the shaft as required, when the cavity is prepared. Prepares gold No. 6, non-adhesive, cut in strips $\frac{1}{2}$, $\frac{1}{4}$, $\frac{1}{8}$ sheets, folded flat into squares; takes one that will fill $\frac{1}{3}$ cavity, forcing it into the bottom of the cavity, next or under the gum, then fills pockets as retaining points, leaving gold projecting beyond the cavities so as to make flush in the last layers or blocks, uses annealed gold, which makes a smooth and harder surface, with serrated condenser, removes all fragments of gold between the teeth, files off the rough portions, and condenses again, and then repeats filing and condensing until he has a perfectly smooth and hard surface, which is finished with a smooth burnisher, soap, &c.

Dr. DRIGGS on preparation of cavities had nothing to say; had for seven years endeavored to put in fillings with the ordinary rope but in two classes never could be successful, viz: in large crown and large proximate cavities. For two years has used cylinders with very much greater success; proceeds as Dr. Rogers does, but uses No. 4 foil, thinks No. 6 too heavy, except for very large crown cavities, taking very great care that the cylinders are not displaced, or that they do not recede under the instrument, fills nearly full, leaving a well shaped cavity near the surface, which is filled and finished with crystal gold foil, leaving a smoother and harder surface than can be done otherwise, thus for approximate cavities; in crown cavities fill full with cylinders, then force the instrument into the center, and fill in a wedge. Crystal foil in contact with the walls of the cavity is inadmissable, except with mallet. Nonadhesive foil adapts itself more closely; related some experiments in which crystal gold plugs were displaced in burnishing, though in a perfectly solid mass, the filling being a unit, perfectly hard; never could use adhesive foil satisfactorily alone, but in conjunction with non-adhesive, using non-adhesive against the walls of the cavity, filling center and surface with the crystal gold, or adhesive foil, uses it folded, taking but three layers at a time, so as to condense *through* each time.

Dr. JUSTICE always prepares cavities as well as he can, to remove all decay, uses Abbey's No. 6, in strips, finds satisfactory results; recently saw some of his first fillings, made years ago; still perfect, never used crystal or adhesive foil.

Dr. E. J. PECKOVER: is a young practitioner; had but little experience, was taking advantage of this meeting to learn; uses Abbey's No. 6 with Leslie's adhesive foil, folded into strips and cut into blocks, packing the non-adhesive foil against the walls of the cavity, finishing center and surface with adhesive; cavity being of course prepared properly with suitable retaining points.

Dr. GRANT had never taken part in discussions, came to learn the experience of old members, wished to profit thereby, was much gratified with what had been said, and will adopt some of the practices described; commonly used ropes of Abbey's No. 4 or 6 adapted to size of cavity, rolled loose; concurrred with Dr. Driggs in the advantages of lamina rollings, as it packs better in folds; spoke of a large proximate cavity in the central incisor, in which it was difficult to retain the gold; did so by placing a wedge between, and using it as if it were the walls.

Dr. TALBERT during the past three or four years' practice, used gold foil No. 4 and 6, of different manufactures, folded with small folds into strips; used Abbey's until recently, as received from the maker; tried crystal gold foil, but did not like it, more recently used ropes of Abbey's No. 4 annealed, found much difficulty, giving much more labor, but repaid by good results; two years ago adopted the annealing of Abbey's No. 6, finds it adhesive, sufficient for all purposes, more plastic and more easily controlled than any other; cut into strips as required for cavity, cutting into strips of $\frac{1}{2}$, $\frac{1}{4}$, $\frac{1}{8}$ sheets, rolling into rope, and pack from end of rope, pressing fold upon fold with rough pointed pluggers, filling up as described, and filling and finishing pockets and depressions last.

Dr. Stone said he commenced filling teeth in early life and had been at it a long time; that although apparently a young man, he was nevertheless old in filling teeth, there are so many varieties of temper, disposition, etc., that it is difficult to do uniform work; had endeavord to be honest to his patients; if all are candid doubted whether at all times we do work just in the best manner we are capable of; there are many difficulties to overcome. At the door meet patients in as cheerful and cordial manner as possible; by this putting patients perfectly at their ease overcomes many difficulties, which would otherwise occur. Commences with the tooth which gives least pain; after filling one or two, proceeds with whatever is diseased. Goes at it first to cut down walls, so as to give strength enough of wall and make free access; uses wedged shaped instruments of all sizes, whether large or small; still uses the flat point some, as thin as a knife blade, after cutting down with burs, chisels, &c.; then takes a little scoop shaped instrument and glass and examines carefully, to remove every particle of decay that may be chipped off, so that no loose particles remain in the bottom; others differ in practice. Having prepared cavities, regardless of shape, so that it is irregular, having strength of wall and freedom of access, the edges of the wall should be smoothed up, so as to give no opportunity for chipping off by pressure; having all prepared he cuts the gold into strips, folds up and twists into a loose springy rope, takes substance enough on point of instrument to form a base in the bottom, then in folds packs against the sides, walling cavity all around, just as a barrel is sustained by its own staves; begins next to the gum, and packs to the top beyond the margin; uses a good amount of force; after filling perfectly full, wedges off in different directions; then fills in the cavities, leaving it to project; if any points are not full, drill out and fill in a piece of gold just as if it were a separate cavity, condense with an oval pointed plugger with large handle, dress up with file and burnisher; then with a piece of wood and prepared chalk, or pumice, this will cut rapidly and expose any imperfections; having all these corrected, go on and dress down with files, hones, &c., until you have a perfectly smooth surface.

Dr. STONE has a great variety of polishing and finishing instruments which he invites the members to call and examine at his office and of which he also promises to furnish patterns, to have made for sale in depots.

AFTERNOON SESSION—APRIL 25TH.

*Second Topic—Fang Filling.*

Dr. ROGERS: For a number of years all fang filling was satisfactory, now cautious in selecting only favorable cases; said he destroys nerve with arsenious acid, being careful to let it remain *only* long enough to destroy the pulp, immediately removes nerve, and requires patient to suck; introduces chloride of zinc, *deliquesced*, not dry, examines, and if sore, repeats injection of zinc; prepares fine pure wire and wrapped with cotton dipped into chloride of zinc, then uses in the fang like a pump or the piston rod of a syringe, until by the movements of patient is satisfied that it is at the point of fang; dismisses patient; cavity opened next day, if not inflamed fills fang and crown; thinks fangs *clogged* with creosote induces inflammation.

Dr. FOOTE thinks the reason given for using chloride of zinc, is the reason he would *not* use it, it being a caustic; eschews all escharotics, and fills a tooth as soon as he satisfied that all particles of nerve or tissue are removed, that can be; but as small particles may be attached in crevices, therefore, after washing clean and stopping blood, for the purpose of rendering what remains insoluble, introduces a little creosote or tannin, then takes fine broaches hardened toward the point, point cut off, ground smooth, and uses as a plugger; takes foil No. 2, winds on the broach, and introduces to the point of the fang and condenses, then adds gold with larger points and fills the fang full; if patient is fatigued let him rest, otherwise proceeds and fills crown also. Sometimes it is very difficult to reach the point of fang, uses a drill made from piano wire, with point shaped like half of an acorn, but very small.

Dr. McClelland fills a great many teeth in the fangs; three or four cases a week; can't say what proportion are lost; don't believe it is more than five per cent. of dead teeth. After finding nerve dead in tooth, first removes all soft tissue with a hardened broach, as near the point as possible; don't try to get through but occasionally does, uses broach, until cotton ceases to be stained; frequently uses hickory wood, in place of broach, and finds it valuable, if tooth is not especially sore; places a pledget of cotton saturated in creosote into the fang, seals with wax and lets it remain three or four days, then fills; but never fills until after it will stand a temporary filling without producing soreness; always fills fangs with gold, in small cylinders made on point of broach; never enlarges fang cavity; only cleanses and clears it, uses square drills beveled on one side.

Dr. Talbert has done almost every thing that has been done, and one thing never will do again; has filled fangs with gutta percha supposing the plastic nature of material would allow him to do it better than with gold, but will do so no more, but *with gold only.*

Dr. Driggs had not much to say, although he performed many operations of the kind; but in candor selects only favorable cases, viz: incisors and cuspids; rarely bicuspids and molars; because of the difficulty of cleaning the fangs or reaching the point; attempts to clean the fang as well as possible; wants to see in; can not be satisfied by feeling, but in some cases has been satisfied to fill only part way; or rather not satisfied that the point was reached.

Dr. PECKOVER prior to the meeting of the American Dental Convention at Cincinnati, did not attempt to fill fangs; has done so since, but occasionally failed, and teeth had to be extracted; has had patients call, who had teeth filled by others, which had to be extracted, does not believe the point of fang can be reached. Dr. Stone, Dr. McClelland, Dr. Rogers and Dr. Foote's remarks express his views; creosote introduced to the point of fang either destroys the remaining fragment of nerve, if any, or else congeals it, or stimulates the absorbents so as to take up all; makes no application to destroy pulp, but removes with barbed broach; never fills as long as there is any appearance of blood, or any secretion remains.

*Third Topic—Alveolar Abscess.*

Dr. ROGERS has less experience in that than almost any other branch; met a case of front incisor large cavity, very offensive, tooth dead, introduced a probe, cut the sac, followed with nitrate of silver, followed again by chloride of zinc, and in a short time had the satisfaction to see it apparently perfectly healed; very careful not to promise to cure abscess.

Dr. TALBERT drills right through the point of the fang, into the abscess, when it can be done; when not, then through the process to the point of fang, then treats with creosote, until healed.

Dr. GRANT felt much solicitude; read much that had been written, and heard discussions; treated many cases, one a fang of lateral incisor having been filled, abscess formed, filling came out; had been out about two years when application was made; undertook the case with doubt and apprehension; first cleaned fang to apex, lanced as described by

Dr. Talbert, and introduced creosote, repeated creosote several times in three weeks, perfectly healed, and tooth sound; cleaned again and treated with creosote and filled with gold; it is still in good condition. After nerve canal has been filled, and fears are entertained of abscess ensuing, makes application of horse radish on the gums, which calls the inflammation to the surface, protecting the mouth of course; used the same frequently in cases of pain (tooth-ache); after teeth are filled.

Dr. McClelland uses a remedy which he thinks is as good, and more convenient of use; chloroform on a pledget of cotton, enveloped in dry cotton, so as not to blister, protects mouth with tin foil.

Dr. Foote uses electricity.

Dr. Driggs uses creosote in the treatment of alveolar abscess, and can in a few days heal up the external aperture; but thinks other treatment must be resorted to, to remove the cause, it being internal.

Dr. Justice's treatment similar to Dr. Driggs, Grant and McClelland's.

Dr. Peckover uses a fig in the formative process of abscess.

Dr. Stone thinks alveolar abscess is nothing but the destructive process of a dead nerve; nature the meanwhile trying to throw it off. The vessels become expanded and the orifices become engorged. Treat the tooth, remove all the pulp, if any remains, wash out all the pus, and probe through the fang into the socket; inject creosote and it heals like any other sore; found for three weeks, every day, some discharge; continued to inject a little creosote every day until the sac was entirely destroyed. As long as any particle of the sac remains, it will be formed again, by inflammation, even after the filling.

*Fourth Topic—Mechanical Dentistry.*

Dr. Foote illustrated the modus operandi of preparing casts, arranging the teeth and obtaining the articulation for the Vulcanite work.

Toland explained the relative merits of the different materials for Vulcanite work, viz: the rubber, coralite, and the Amber base.

*Fifth Topic—Partial Dentures.*

Drs. Peckover, Sen., Peckover, Jr., Foote, Driggs, and others, gave some experience of a valuable nature, resulting in the conclusion that the Vulcanite base is particularly applicable, on account of its perfect adaptation, and congeniality to the mouth and teeth.

*Sixth Topic—Articulation of the Jaws for Full Dentures.*

Dr. Rogers after producing plates for upper and lower, places them in the mouth with wax between them, to procure the draft of about the space to be occupied, places small wires upon the ridge that may mark the proper occlusion of the jaws; requires the patient to open and close the mouth several times to see if the occlusion is correct; then removes from the mouth; then carve out the wax to accommodate the teeth, in the place indicated; arrange the upper teeth first, as it is more necessary to have the proper fullness of contour in the upper than in the lower.

Dr. Foote pursues the same course except the use of pins, but places only a few teeth on the plate at first, especially if the patient is convenient, then adjust the balance.

Dr. Stone remarked that Dr. Hullihen had great celebrity in this matter; he always fixed the bicuspids first, and finished from that point.

Dr. Talbert: if you tell the patient not to put out the chin they are more likely to protrude it than otherwise. He has two modes of obtaining a correct articulation; first, let the patient be unguarded and keep him engaged in conversation, until they close naturally, then mark the place. If this does not answer, request the patient to swallow the saliva in the mouth, which can not be done without closing the mouth in a natural way.

Dr. DRIGGS was formerly guided by the swallowing process, but has met with several cases in which it has utterly failed; but now depends upon conversation.

Dr. McCLELLAND finds difficulty in getting the patients to close the mouth naturally; in partial sets asks them to close the jaw teeth, which brings them about right.

With wax in the mouth, and muscles in repose, marks the wax for the length of the teeth, about the length of the upper teeth or a little longer; then requires them to protrude the chin, then draw it back, then to open and close as they will generally, close correctly; then marks the median line and position of the teeth; commences at the bicuspids, fastening the teeth to the plate by adhesive wax, then try in the mouth.

Dr. FOOTE had a case in which he got the lower jaw too far back. It was a lady who had been without teeth for a long time.

Dr. MOFFIT differs from others in getting the jaw back; never could get any too far back; after several efforts in requiring patient to open and close; if not right presses against the lower jaw, with one hand, the other on the head, and forces the jaw back.

Dr. JUSTICE first places a piece of wax in the jaws and lets the patient close them, biting; placing a small stick between the wax to bite upon.

Dr. PECKOVER has nothing especially new; pretty much the same as others; when he finds difficulty in getting a patient to close correctly, places teeth in the mouth and lets her walk about the room for five or ten minutes, look in the glass, &c., and then seats her again and makes them as they are; sometimes places pins in the upper wax requiring them to close so as to pierce the lower.

*Seventh Topic—Miscellaneous.*

Dr. JUSTICE relates a case of irregularity very interesting, asking the opinions of members as to the course to be pursued.

The discussions here became of a conversational character, of questions and answers, highly entertaing and interesting, eliciting some curious and valuable information, important to the profession.

Gentlemanly courtesy, liberality of sentiment, freedom of expression, charactesized the meeting; indeed all seemed to act upon the principle of freely give and freely shall you receive. They seemed to feel that it was good to be here.

There seems to be no doubt but that this movement has fallen into good hands, and is calculated to do much good. A handsome representation was present; but none should have been absent. T.

